# Natural-history traits suspected behind interspecific variations of bark- and wood-boring beetles in response to trap size and design

**DOI:** 10.1038/s41598-025-16511-6

**Published:** 2025-10-01

**Authors:** Emilio Caiti, Séverine Hasbroucq, Jean‑Claude Grégoire

**Affiliations:** 1 Unit of Evolutionary Biology & Ecology, Université libre de Bruxelles, CP 160/12, 50 Av. FD Roosevelt, Bruxelles, 1050 Belgium; 2 Spatial Epidemiology Lab, Université libre de Bruxelles, CP 160/12, 50 Av. FD Roosevelt, Bruxelles, 1050 Belgium

**Keywords:** Monitoring, Trapping, *Ips typographus*, Pest management, *Pityogenes chalcographus*, *Trypodendron* spp., Forestry, Entomology

## Abstract

The performances of traps used for pest monitoring are not well understood. Variable trap sizes and shapes make cross-comparisons difficult. We tested with four Scolytinae species (Coleoptera, Curculionidae) a set of traps of the same model (fan-traps) in four sizes (310, 536, 832.5, 1,200 cm^2^), and a larger trap with another design (a four-vane trap, 4,500 cm^2^). A significantly linear relationship was observed between trap sizes and catches for *Pityogenes chalcographus*, but the catches of *Ips typographus*, *Trypodendron lineatum* and *T. signatum* tapered off. In the literature, comparable tapering-off effects were observed with *Xyleborus glabratus* (Scolytinae) and *Agrilus planipennis* (Coleoptera, Buprestidae), but a significant linear response with increasing trap size was observed with *Trypodendron lineatum* and *T. rufitarsus*. In another test, beetle landing behaviour was measured on a 1 m^2^ glued panel baited with lineatin (the *Trypodendron* spp. pheromone) and ethanol (a host kairomone). The landing densities of *Trypodendron* spp. did not increase with the distance to the attractants whilst *Anisandrus dispar* landed in very significantly higher densities far from the attractants. Species using pheromones to locate mate and host appear to land closer to the source of attractants than species responding to kairomones for locating a suitable host.

**Supplementary Information:**

The online version contains supplementary material available at 10.1038/s41598-025-16511-6.

## Introduction

The routine monitoring of alien or native pest has become a widespread and often compulsory procedure in plant protection^1–5^. Trap networks have been deployed at national scales (e.g^6^. or even worldwide^7^. Trapping procedures and techniques applied to bark- and wood borers have been analysed and improved in different parts of the world (e.g^8–17^. Various factors can influence trapping efficiency. Sweeney et al. (2023)^18^ and Dodds et al. (2024)^12^ divide them as extrinsic and intrinsic. Extrinsic factors depend on the environment, including location^19,20^, the surrounding landscape^21^ or meteorological variables^22^. Intrinsic factors include characteristics of the trap itself. Some have been tested, for example the position of the lures^23^, the use of lubricants, an anti-escape ring^24^, wet versus dry retrieval cups, or trap colour^10^. Cavaletto et al. (2020)^25^ also showed that trap colour significantly influences both the richness and abundance of Buprestidae, Scolytinae and Cleridae. However, apart from a few exceptions, the influence of trap size on the efficacy of monitoring (abundance and diversity of potential pests) has not been systematically investigated so far. To our knowledge, only four studies provide sufficient data (> 2 sizes) for analysing a trend. Brar et al. (2012)^26^ recorded the response of *Xyleborus glabratus* Eichhoff (Coleoptera, Curculionidae, Scolytinae) to a host primary attractant, manuka oil, in funnel-traps with increasing numbers of funnels (4, 8, 12, 16). Francese et al. (2013)^9^ analysed *Agrilus planipennis* Farmaire (Coleoptera, Buprestidae) responding to the visual attraction of green traps with 4, 8, 12, 16 funnels. Hoover et al. (2000)^27^ and Lindgren et al. (2000)^28^ reported catches of three *Trypodendron* species (Coleoptera, Curculionidae, Scolytinae) in traps with 4, 8, 12, 16 funnels baited with lineatin. In addition, three studies to our knowledge compared two trap sizes. Francese et al. (2010)^8^ compared the response of *A. planipennis* to sticky traps with interception areas of 1,600 cm^2^ and 6,400 cm^2^ and found that the numbers of beetles caught increased significantly with trap size, but that landing density remained constant; Miller & Crowe (2009)^29^ compared the responses of several beetles species including Curculionidae and two Cerambycidae species to traps with 8 or 16 funnels baited with ethanol and α-pinene, and found diverging results according to each species trapped. Branco et al. (2004)^30^ showed that larger sticky traps (900 cm^2^ instead of 225 cm^2^) significantly caught more males of two *Matsucoccus* spp. (Hemiptera, Matsucoccidae). Miller & Crowe (2009)^29^, using traps with eight and 16 funnels showed divergent trap size preferences depending on species; for example, *Arhopalus rusticus nubilus* LeConte (Coleoptera, Cerambycidae) responded in significantly higher numbers to the 16-funnels traps, and *Hylobius pales* Herbst (Coleoptera, Curculionidae) was caught in larger numbers in the eight-funnels traps.

Trap size influences costs as well as all the logistics aspects of monitoring campaigns (shipment, transportation, establishment, trap collections) and, therefore, a compromise between size and performances must be found. Here we analyse how trap size influences the catches of different species and, in this context, how the behaviour of some of them can explain their response to semiochemicals. We present the results of experiments specifically designed to match trap performances against size, using a standard design, the *fan-trap*^31^, that can easily be scaled up, and by further adding for comparison with a yet larger size, a standard, commercial four-vane trap (Econex, Murcia, Spain). We then compare our results with those of the previous studies listed above that compared more than two trap sizes. Finally, based also on additional results from a previous experiment, we discuss how each species’ responses to semiochemicals (pheromones signalling conspecifics *versus* kairomones signalling a suitable host) could affect landing behaviour, and how this translates into adequate trap size.

## Materials and methods

### Gedinne (2023)

**Fig. 1:**
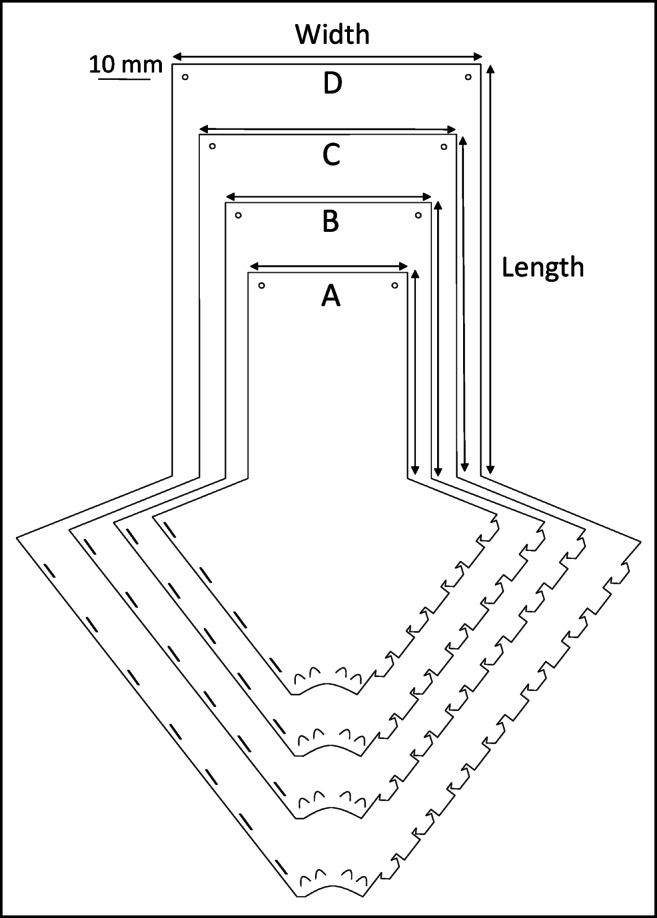
Combined blueprints of the different *fan-traps* sizes. The dimensions of the interception panels are given in Table 1.

Four different *fan-traps* of increasing sizes (respective interception areas: 310, 536, 832.5, 1 200 cm^2^) were compared (Fig. 1; Table 1), all based (materials, design and construction) on an original blueprint described by Grégoire et al. (2022)^31^. As the interception area increased, the arrow-shaped part corresponding to the conical collector had to be modified to allow the interception panel to always remain flat after the folding and fixation on a support. These *fan-traps* were also compared to Teflon-coated four-vane *Econex Mini* traps (Sanidad Agricola ECONEX, El-Siscar-Santomera, Murcia, Spain) (vane: 50 cm x 11,25 cm; total vane area: 4,500 cm^2^; cylindrical area: 3532.5 cm^2^).

**Table 1:** Measure of the interception panel for each *fan-trap* size (a to d).

Fan-trap	Width (mm)	Length (mm)	Interception area (cm^2^)
A	155	200	310
B	200	268	536
C	250	333	832.5
D	300	400	1,200

**Fig. 2:**
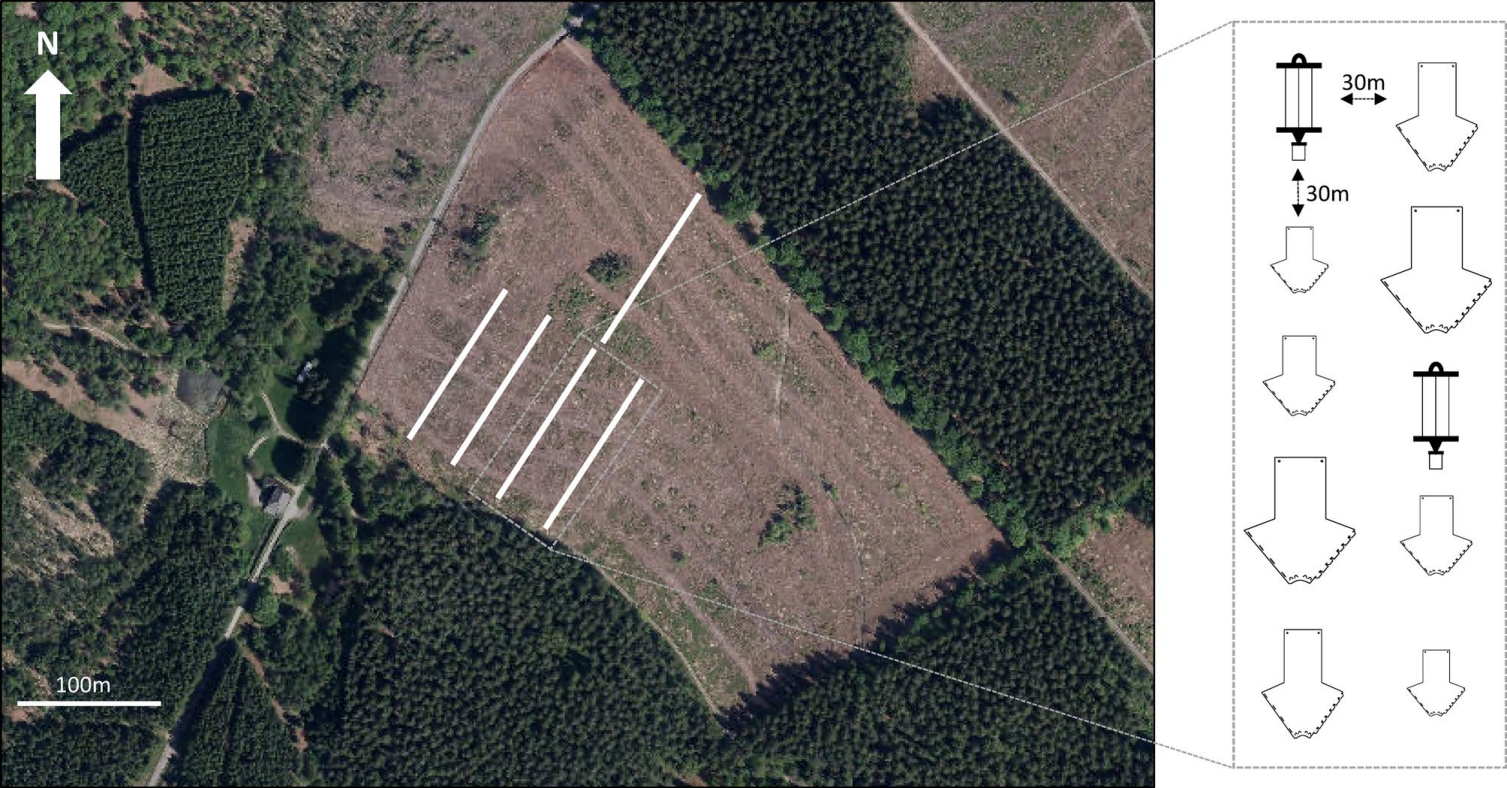
Experimental design and position of each line in the clear-felled area in Gedinne. Original picture generated on WalOnMap, SPW (2023)^34^ modified.

**Fig. 3:**
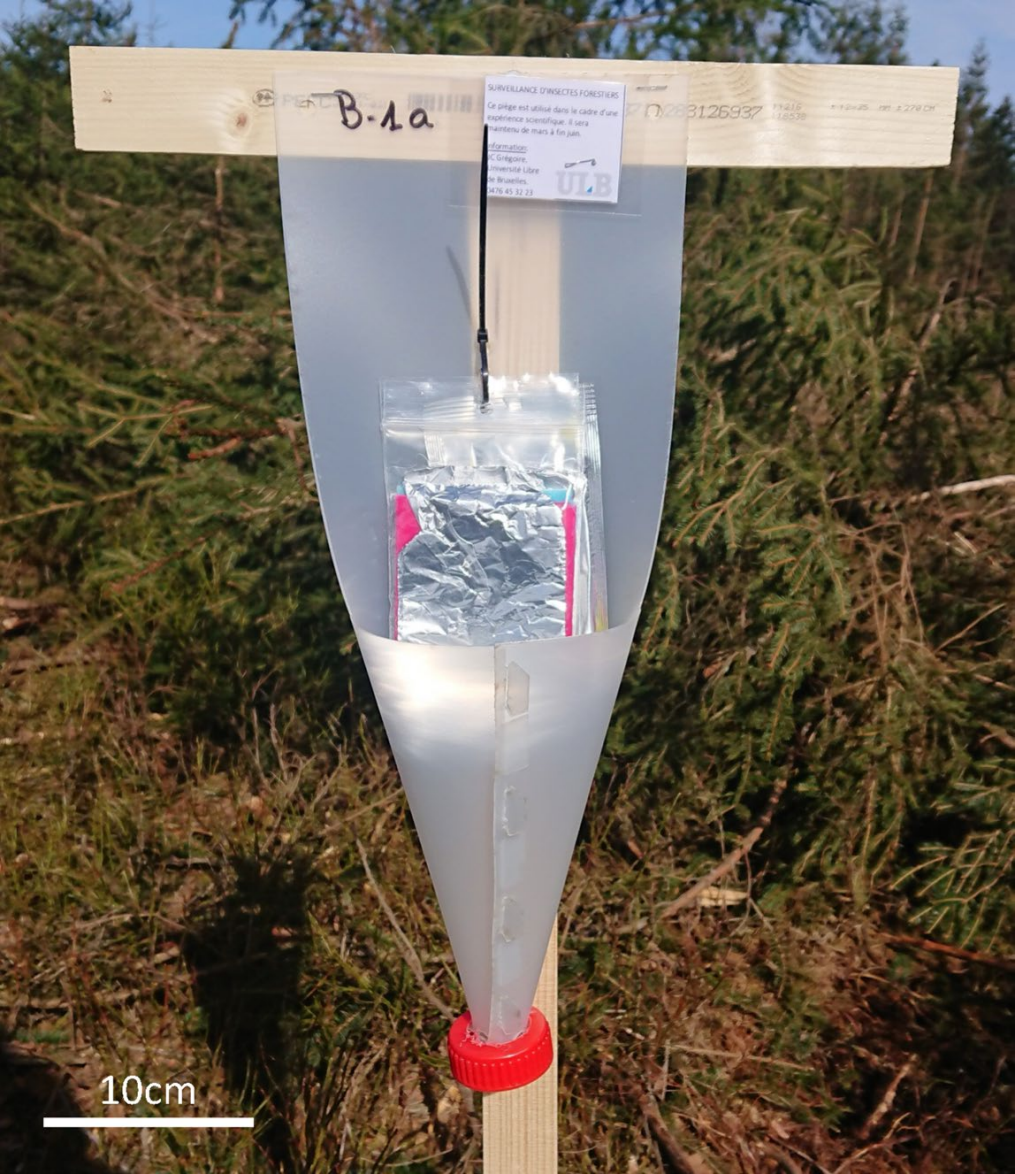
*Fan-trap* (size B) on a wooden support (Gedinne, 2023).

The experiment was set up in Gedinne, province of Namur, Belgium (49.960657, 4.821336). Five lines of traps, oriented NE and and separated by 30 m from each other were spread out in a one-year-old clear-cut area bordering Norway spruce and Scots pine stands (Fig. 2). Each line comprised four *fan-traps* (one of each size A, B, C, D and one *Econex Mini* four-vane trap). The *fan-traps* were supported each by a T-shaped wooden structure made of one vertical (130 × 3,5 × 1 cm) post supporting a (32 × 3,5 × 1 cm) horizontal piece of wood, to which the upper side of the trap’s interception panel was stapled (Fig. 3). The *Econex Mini* traps were set up suspended to a tripod 180 cm high (*Trinet* trap stand, Witasek, Feldkirchen, Austria). All traps, including the suspended four-vane traps, were hanging ca. 50 cm above the ground, and distant by 30 m from each other. The traps were set up on 16 March 2023, visited on 30 March,13 April, 27 April,16 May, 25 May and 7 June 2023, when they were removed. On each visit, the order of the traps within each line was modified by drawing at random a piece of paper among five, one for each type of trap. Every trap was baited with a semiochemical dispenser, placed in the centre of the interception panel of the *fan-traps*, or in the middle of the external edge of one of the panels of the four-vane traps. The dispensers were renewed each time the traps were serviced. Two different lures were successively used during the season, to attract different Scolytinae species: three *Trypodendron* species, *T. lineatum* (Olivier), *T. signatum* (Fabricius), and *T. domesticum* (L.) from 16 March to 16 May, and *Ips typographus* and *Pityogenes chalcographus* between 16 May and 7 June. The *Trypodendron* spp. lures on each trap consisted in a *Lineatin Kombi* dispenser (Witasek, diffusion rate 50–100 mg/d) and two ziplock bags (12 × 8 cm x 50µ) containing each 50 ml of denatured ethanol (96%) impregnating four 10 × 7 cm pieces of viscose tissue wrapped in aluminium foil (anti-UV protection). The lures for *I. typographus* are constituted of a ziplock bag (6 × 8 cm x 50µ) encasing a cellulose wick impregnated with 80 mg of (S)-*cis*-verbenol (Merck, 95% purity) diluted in 2 ml of 2-methyl-3-buten-2-ol (Merck, 98% purity) with a diffusion rate of ca. 60 mg/d for 29 days) as described by Inward et al. (2024)^32^. The diffusion rate of the ethanol dispensers remained between 500 mg/d and 300 mg/d during each two-weeks period between two servicings (supplementary material: Fig. 1). The collecting bottles of all traps contained 50% propylene glycol (1,2-propanediol, Sigma-Aldrich) diluted in water, as a preservative. At every service, the insects were retrieved from the collecting containers and transferred in ethanol (70%) for identification at a later stage, under a binocular lens Leica MZ6 (15,75x-100x), based on the taxonomic keys of Balachowsky (1949)^33^.

### Bertrix (2002)

**Fig. 4:**
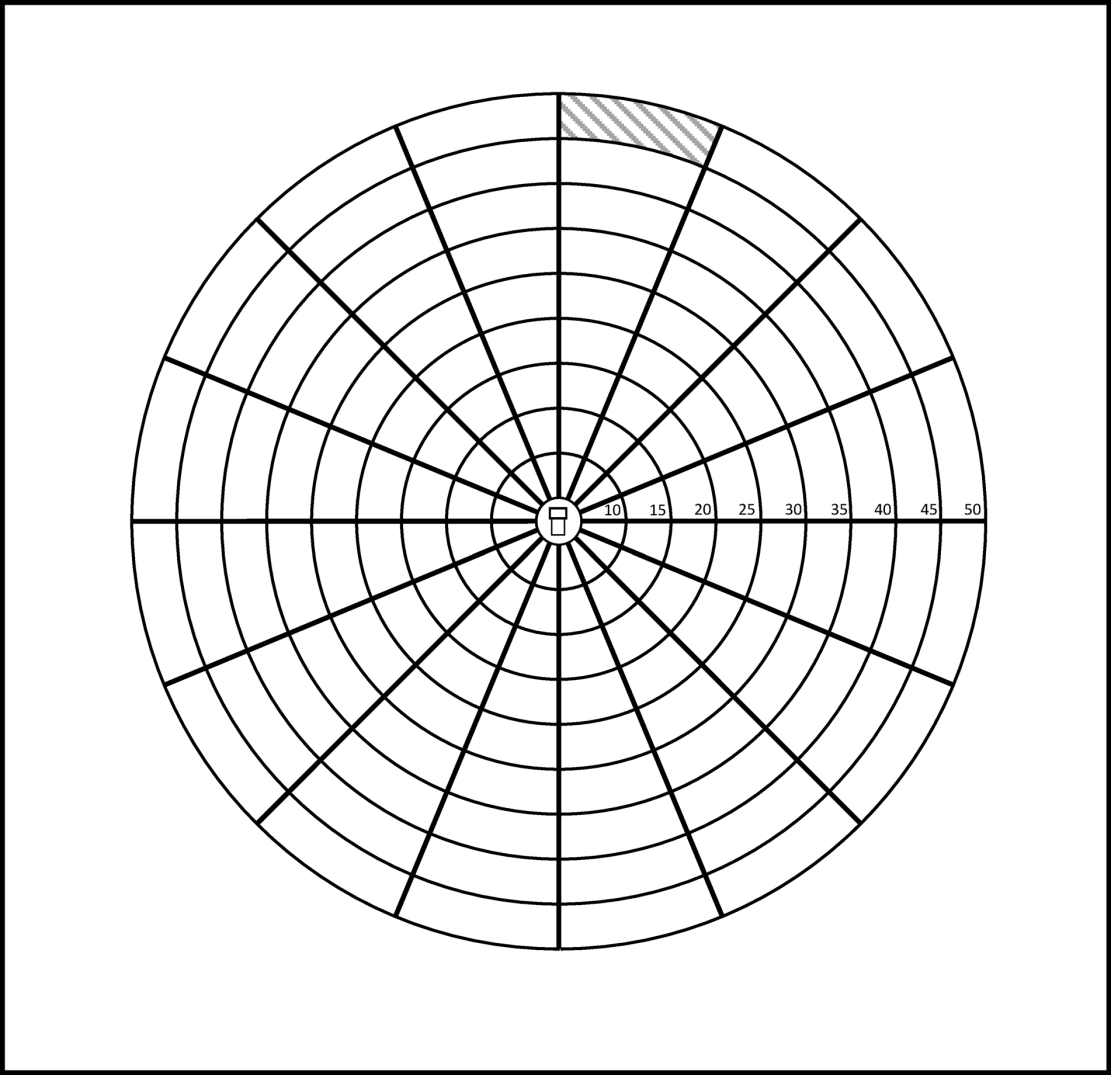
The glue-trap used in Bertrix (2002) (size: 100 × 100 cm) with one half-quadrant cell on one ring hatched.

To complement the interpretation of the results obtained in Gedinne with *Trypodendron* spp., we used past, unpublished recordings of their landing behaviour at growing distances from a lure, obtained in another context in 2002, in Bertrix, prov. of Luxembourg, Belgium (approx. 49.822; 5.271). Data obtained with another species, *Anisandrus dispar* Fabricius (Coleoptera, Curculionidae) were added to the analysis. A 100 × 100 cm crystal polystyrene panel was positioned vertically between wooden stakes, 50 cm above the ground in a mature beech stand, and coated with glue (Phero Tech, Inc, Vancouver, British Columbia, Canada) on one face. To facilitate analysis, concentric circles were marked on the panel every 10 cm from the centre and divided into eight half-quadrants (Fig. 4). The panel was equipped with a lineatin ((1*R*,2*S*,5*R*,7*S*)-1,3,3-trimethyl-4,6-dioxatricyclo[3.3.1.0^2,7^]nonane) dispenser (50 mg/d; PheroTech) and an ethanol dispenser (open polystyrene bottle containing 50 cc of denaturated ethanol), both suspended in the middle of a 10 × 5 cm window cut at the centre of the panel. The device was set up on 15/05/2002 and removed on 23/05/2002.

#### Comparisons with the literature

Our findings were compared to those of a study by Brar et al. (2012)^26^ on the response of *Xyleborus glabratus* Eichhoff (Coleoptera, Curculionidae, Scolytinae) to a host primary attractant, manuka oil, in funnel-traps with increasing numbers of funnels (4, 8, 12, 16). As well as those of a study by Francese et al. (2013)^9^ on *Agrilus planipennis* Farmaire (Coleoptera, Buprestidae) responding to the visual attraction of green traps with 4, 8, 12, 16 funnels. And finally, to those of Hoover et al. (2000)^27^ on *T. lineatum* in traps with 4, 8, 12, 16 funnels baited with lineatin and of Lindgren et al. (2000)^28^ on *T. rufitarsus*) responding to the same array of funnel-traps baited with lineatin.

### Data analysis

#### Global comparisons

To compare the total catches of each species among different traps, including the four-vane traps, an ANOVA test was used for *P. chalcographus* and *T. signatum* followed by Tukey’s Honestly Significant Difference (HSD) test to identify specific differences in means between trap types. For *I. typographus*, *T. lineatum* and *T. domesticum* a Kruskal-Wallis test was applied, as homoscedasticity variances and normality were not always respected, followed by a Wilcoxon rank sum with the Benjamini-Hochberg adjustment for multiple comparisons.

#### Relationship between fan-trap size and catches

The experiment was based on five blocks (here lines), with the treatment randomised within each block. The proportions of catches (the total catches of one *fan-trap* model against the total catches of the four *fan-traps* models along each line) were used for the analyses that focused only on the *fan-traps*.

In these analyses, *Trypodendon lineatum* and *T. signatum* showed similar profiles (shape and value), so they were grouped for conciseness. Due to the low abundance of *T. domesticum* (total catches: 303 individuals), this species was excluded from the analyses.

#### Further increasing interception area

In a further step, more exploratory because their design differs from the *fan-traps*, Econex four-vane traps were added to the comparison to explore the responses to an even larger interception area. Again, the analysis focused on proportions of catches within each line.

#### Comparisons with the literature

This comparison across experiments with different traps and genera was made possible by using size ratios (trap size : size of the smallest trap in the compared set) and capture ratios (total trap capture : total capture of the smallest trap in the compared set). The data used are available in the supplementary document (Table 4).

#### Landing behaviour of trypodendron spp. And Anisandrus dispar

In the glue-trap experiment, we analysed the number of catches, as well as the landing density, against the distance from the lures at the centre. The landing density corresponds to the number of individuals captured in each half-cell of each ring divided by the area of this half-quadrant cell. In this experiment *T. lineatum* and *T. signatum* were also grouped.

The ANOVA and the analyses on landing behaviour were performed using R Studio software (ver. 4.3.2) with the specified packages stats and ggplot2^35,36^. The regressions were analysed using Microsoft Excel ver. 16.89.1.

## Results

### Global comparison

**Fig. 5:**
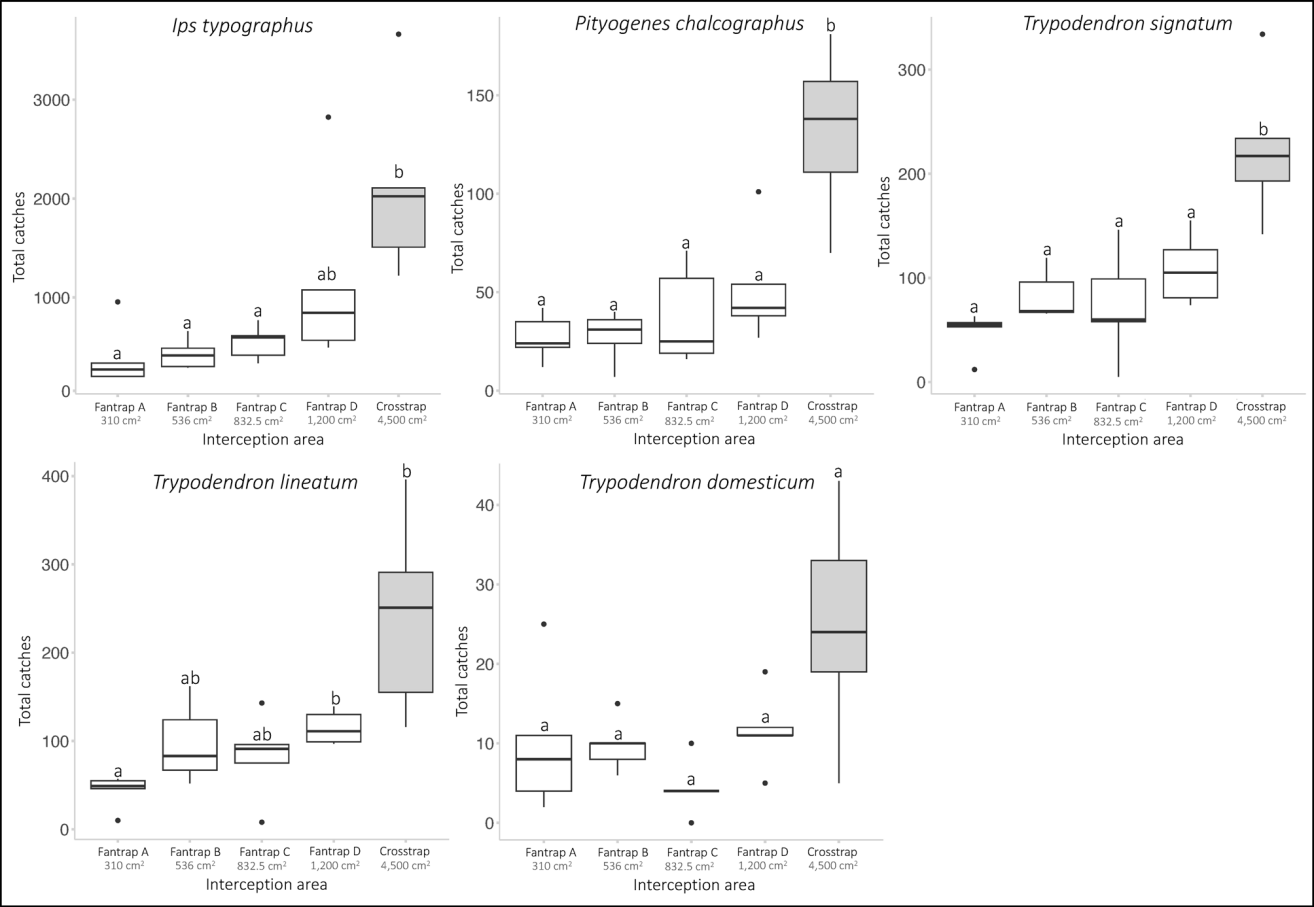
Box plots of the total catches of every trap type on each line (*N* = 5), by species. The different *fan-trap* catches (A, B, C and D) are represented in white boxes and the four-vane trap catches in grey boxes. Plots followed by the same letter are not significantly different at $\:\alpha\:$ = 0.05.

A total of 30,135 beetles were caught: 23,234 *I. typographus*, 1,380 *Pityogenes chalcographus*, 2,708 *T. signatum*, 2,915 *T. lineatum* and 303 *T. domesticum*. The catches of each species are given in the supplementary material (Table 1). The global trapping results for the five trap models are presented in Fig. 5. Overall, there was a significant difference between traps for every species: *I. typographus* (Kruskal-Wallis: chi^2^_4_ = 15.41; *p* = 0.004); *P. chalcographus* (Anova: F_4_ = 13,38; *p* < 0.001); *T. signatum* (Anova: F_4_ = 12.63; *p* < 0.001; *T. lineatum* (Kruskal-Wallis: chi^2^_4_ = 15.33; *p* = 0.004); *T. domesticum* (Kruskal-Wallis: chi^2^_4_ = 10.47; *p* = 0.03). There were no differences in catches of the *fan-traps* (*I. typographus*; *P. chalcographus*; *T. domesticum; T signatum*) or little differences (*T. lineatum*) between the *fan-traps* (ABC) and the larger one (D). The four-vane traps, on the other hand, caught more insects in all species, although the catches of *I. typographus*, *T. lineatum* and *T. domesticum* were not significantly different from those of the largest *fan-traps* (Fig. 5; Table 2).

### Comparison with other experiments in the literature

**Fig. 6:**
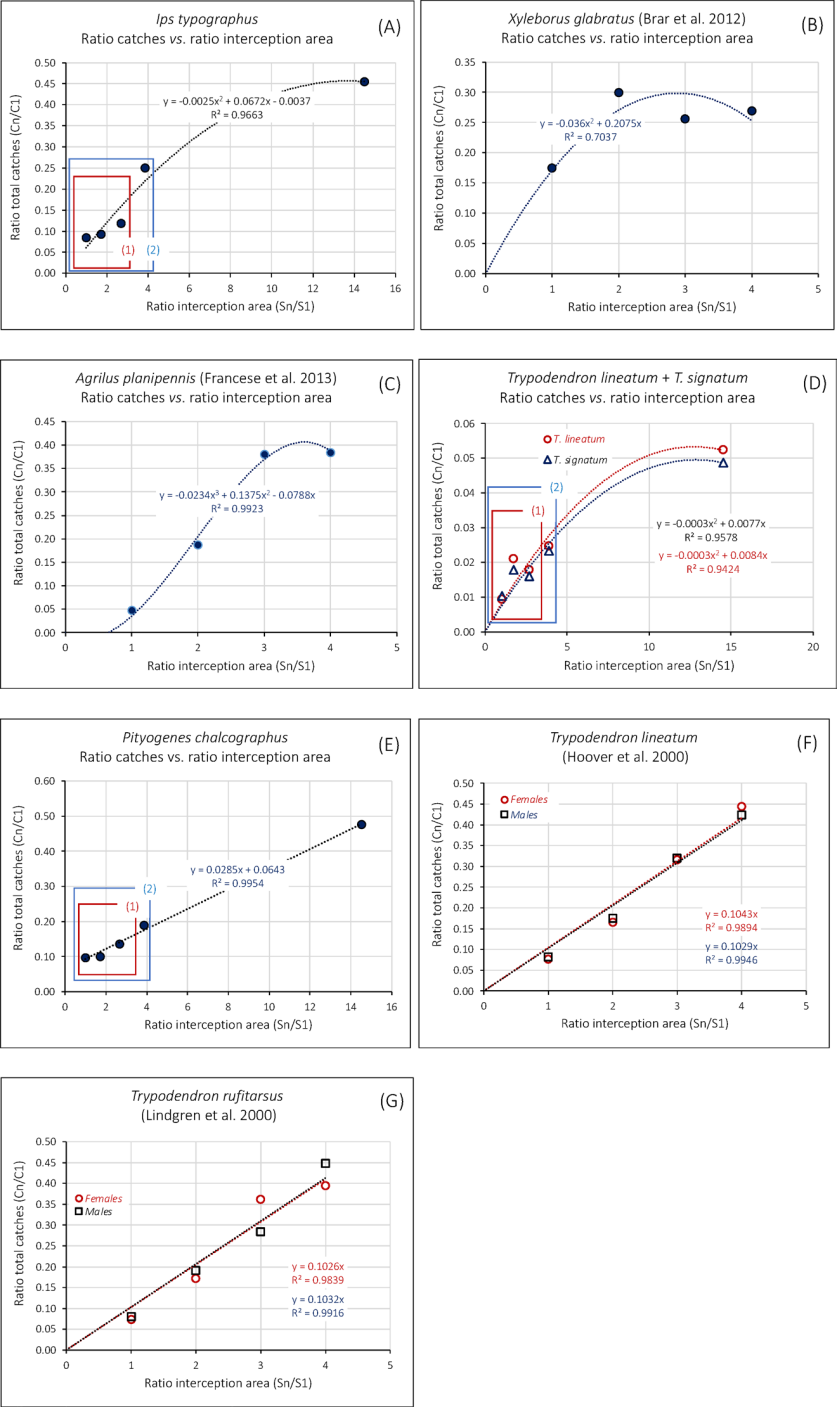
Trends in the response of bark- and wood boring beetle species from our experiments and from the literature. (**A**) *Ips typographus*, this study; (**B**) *Xyleborus glabratus*^26^; (**C**) *Agrilus planipennis*^9^; (**D**) *Trypodendron signatum* & *T. lineatum*, this study; (**E**) *Pityogenes chalcographus*, this study; (**F**) *Trypodendron lineatum*^27^; (**G**) *Trypodendron rufitarsus*^28^. More details regarding the coloured boxes in Fig. 6D and E are provided in Figs. 2 and 3 in the Supplementary material.

Beyond the ANOVA above, a regression analysis allowed us to disclose finer relationships between trap size and catches of the different species compared. Relationship between the ratios of total catches to the ratios of interception areas are represented in Fig. 6. The proportion of total *I. typographus* catches in Gedinne tapers off with increasing trap size (Fig. 6A). This is also observed with the data from Brar et al. (2012)^26^ with *X. glabratus* (Fig. 6B), with those from Francese et al. (2013)^9^ with *A. planipennis* (Fig. 6C), and with the catches of *T. lineatum* and *T. signatum* in Gedinne (Fig. 6D).

On the contrary, the relative catches of *P. chalcographus* in Gedinne (Fig. 6E) continue to grow linearly with trap size, and so do the *T. lineatum* catches reported by Hoover et al. (2000)^27^ (Fig. 6F) and the *T. rufitarsus* catches reported by Lindgren et al. (2000)^28^ (Fig. 6G). In these two last examples, the responses of both sexes are strikingly similar. Table 2 summarises these relationships.

**Table 2:** Summary of model fits for each study.

Study	Equation	*R* ^2^	*p*
*Ips typographus* (Gedinne)	y = − 0.0026 × ^2^ + 0.0693x – 0.0088	0.96	0.034
*Xyleborus glabratus* (Brar et al. 2012)	Y = − 0.036 × ^2^ + 0.2075x	0.70	0.011
*Agrilus planipennis* (Francese et al. 2013)	y = − 0.0234 × ^3^ + 0.1375 × ^2^ − 0.0788x	0.99	0.055
*Trypodendron lineatum* (Gedinne)	y = − 0.0014 × ^2^ + 0.0346x	0.94	0.04
*Trypodendron signatum* (Gedinne)	y = − 0.0012 × ^2^ + 0.0319x	0.96	0.021
*Pityogenes chalcographus* (Gedinne)	y = 0.0285x + 0.0643	0.99	< 0.001
*Trypodendron lineatum* (females) (Hoover et al. 2000)	y = 0.1256x − 0.0641	0.99	0.005
*Trypodendron lineatum* (males) (Hoover et al. 2000)	y = 0.1174x − 0.0436	0.99	0.003
*Trypodendron rufitarsus* (females) (Lindgren et al. 2000)	y = 0.1026x	0.98	0.008
*Trypodendron rufitarsus* (males) (Lindgren et al. 2000)	y = 0.1032x	0.99	0.031

### Response to increasing interception areas: different species, different trends

The response to trap size depended on size ranges and on species. Within the smaller size ranges, all species responded weakly, on a linear manner, to increased sizes, as shown in Figs. 6A (boxes 1–2), 6D (boxes 1–2), and 6E (boxes 1–2). More details are provided in Figs. 2 and 3 in the Supplementary Material. The response of *I. typographus* shows a trend towards tapering off (Fig. 6A), whilst the responses of *X. glabratus* and *A. planipennis* definitely taper off (Figs. 6B and C). The apparent contradiction between our results for *Trypodendron* spp. (Fig. 6D) and those in the literature (Fig. 6F and G) is discussed below.

### Landing behaviour of *Trypodendron* spp. and *Anisandrus dispararound a lure*

**Fig. 7:**
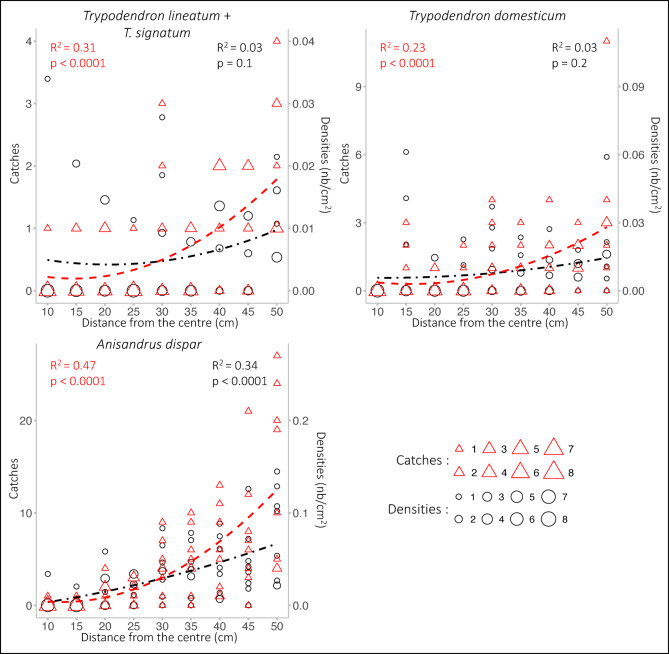
Effect of the distance from the attractants (cm) on the catches (red triangles) and densities of catches (black circles) of *Trypodendron lineatum* + *Trypodendron signatum*, *Trypodendron domesticum* and *Anisandrus dispar* (polynomial fit) on a one cm^2^ plexiglass panel covered with glue, with the lures placed at the center. The size of the triangles and circles is proportional to the catches and densities, respectively.

For each species, a positive relationship was observed between the number of individuals trapped and the distance from the lure (Fig. 7). *Anisandrus dispar*: R^2^ = 0.47; *p* < 0.001; residual standard error = 4.4, df = 69; *T. lineatum* + *T. signatum*: R^2^ = 0.34, *p* < 0.001, residual standard error = 0.8, df = 69; *T. domesticum*: R^2^ = 0.23, *p* < 0.001, residual standard error = 1.5, df = 69.

The changes in the densities of catches, however, varied between species (Fig. 7). No increase was observed with *T. domesticum*. (R^2^ = 0.03, *p* = 0.2, residual standard error = 0.01, df = 69 ) and *T. lineatum* + *T. signatum* (R^2^ = 0.34, *p* < 0.001, residual standard error = 0.008, df = 69), but the landing density of *A. dispar* increase significantly with the distance from the lures (R^2^ = 0.34, *p* < 0.001, residual standard error = 0.03, df = 69).

## Discussion

The conclusions to be drawn from these results are twofold.

*A first outcome is technical*. According to the ANOVA, within a certain range (310 to 832.5 cm^2^), trap size did not influence much the catches of the four species tested, *I. typographus*, *P. chalcographus*, *T. signatum* and *T. lineatum* (Fig. 5). Beyond this size, the catches increased for all species except *T. domesticum* (Fig. 5); this could have been linked both to a higher trap size and to a different trap model (four-vane vs. fan-trap). However, according to the regression analysis illustrated in Fig. 6, the catches of all species in our experiment increased with trap size, in different fashions according to species. So, if a choice must be made between the four smaller trap sizes in our experiment, choosing the smaller, 310 cm^2^ traps, would not affect very much a monitoring campaign targeting the species tested here. On the other hand, choosing the much larger, 4,500 cm^2^, trap model in our tests would increase monitoring performances. Practically, considering other aspects linked to trap deployment (handling time and other human costs, financial cost, convenience), a trade-off should be considered between trap numbers and trap efficacy. Higher trap numbers could compensate reduced performances. An ongoing study on Scolytinae in Belgium (in preparation) showed that when six 310 cm^2^ fan-traps were deployed together with one four-vane trap, they collectively caught more individuals and more species than the four-vane trap. In addition, they were markedly cheaper, required less handling time and were by far easier to transport and deploy.

*A second conclusion relates to the biology of the target species* and offers insights into how trap size (interception area) and, possibly, trap shape, could be adjusted to the behaviour of each species.

*Pityogenes chalcographus* and *Trypodendron* spp. tended to react linearly to size. The tapering-off response of *T. lineatum* and *T. signatum* to the larger traps in Belgium could derive from the wider trap surface ratios in these experiments (1:14.5) as compared to the 1:4 ratios in Hoover et al. (2000)^27^ and Lindgren et al. (2000)^28^, possibly inducing a saturation effect. Figure 3C in the Supplementary material shows that, when the highest interception area ratio is 1:4 as in Hoover et al. (2000)^27^ and Lindgren et al. (2000)^28^, the response of *T. lineatum* and *T. signatum* to increasing sizes in Gedinne was also linear. These species respond to kairomones, either (*Trypodendron* spp.) to ethanol produced by weakened trees, or (*P. chalcographus*) to the pheromones of *I. typographus*, signalling a tree under attack with a crown open to colonisation by *P. chalcographus*. Their target may not, therefore, be a particular point on a tree (an individual producing pheromones) but rather the tree as a whole. These species also respond to their own pheromones (lineatin for *Trypodendron* spp^37^. ; chalcogran and methyl (E, Z)-2,4-decadienoate for *P. chalcographus*^38,39^, and those would allow them to find a mate at close range on a host located via kairomone signals. This is supported by the information provided by the glue-trap experiment (despite its lack of replications), where the *Trypodendron* spp. landed at the same densities at any distance from the lures. In contrast, *A. dispar* landed at even growing densities when distant from the lures. *A. dispar* is a sib-mating species in which only the females fly and colonise new hosts^33^; whilst ethanol would signal a favourable, weakened host, the lineatin in our experiment would indicate the proximity of potential competitors (*Trypodendron* spp.) and could exert a weakly repulsive effect inducing *A. dispar* to settle preferentially far from the source of attractants.

Three other species in our test (*I. typographus*) or in the literature (*A. planipennis* and *X. glabratus*) behaved quite differently, apparently targeting the lures, within certain limits indicated by their declining response beyond a certain range.

*Ips typographus* responded here to pheromones, even though the males tend to create new brood chambers at variable distances from a pheromone source as reported by Toffin et al. (2018)^40^. The females in search for a mate are however more likely to land close to the source. For individuals of both sexes, remaining close to the source might also prove safer when landing on standing, mass-attacked trees that develop induced defence.

*Agrilus planipennis* responded to visual stimuli (the green colour of the traps), and, for this species, a response proportional to trap size could be linked to the increasing distance at which the signal could be seen, with a limit corresponding to the horizon of the visual landscape of the foraging beetles and to the population density within this landscape. *Xylosandrus glabratus* constitutes another puzzling and, so far, unexplored case. Although it responded to host volatiles mimicking the host’s in the study of Brar et al. (2012)^26^, it appeared to focus on the proximity of the source as suggested by its tapering off response. This invasive species has recently colonised new hosts in North America, and a deeper understanding of the complex relationships developed with these new hosts could perhaps in the future clarify how it responds to host kairomones.

The main conclusion to be drawn from this study is thus that increasing our knowledge of the foraging and landing behaviour of bark- and wood boring beetles would both benefit our understanding of their biology and our capacity to survey them.

## Supplementary Information

Below is the link to the electronic supplementary material.

Supplementary Material 1

## Data Availability

The datasets generated during and/or analysed during the current study are available in the supplementary materials.
