# Supplementary material for: Natural-history traits suspected behind interspecific variations of bark- and wood-boring beetles in response to trap size and design
Source: Sci Rep. 2025 Oct 1;15:34272. doi: 10.1038/s41598-025-16511-6 (PMC12488991; doi:10.1038/s41598-025-16511-6)
Supplement: Supplementary file 1 — Supplementary Material 1 [file 41598_2025_16511_MOESM1_ESM.pdf]

Caiti et al. - Supplementary material

Natural-history traits suspected behind interspecific variations of bark- and wood-boring beetles in response to trap size and design

Emilio Caiti; Séverine Hasbroucq; Jean-Claude Grégoire

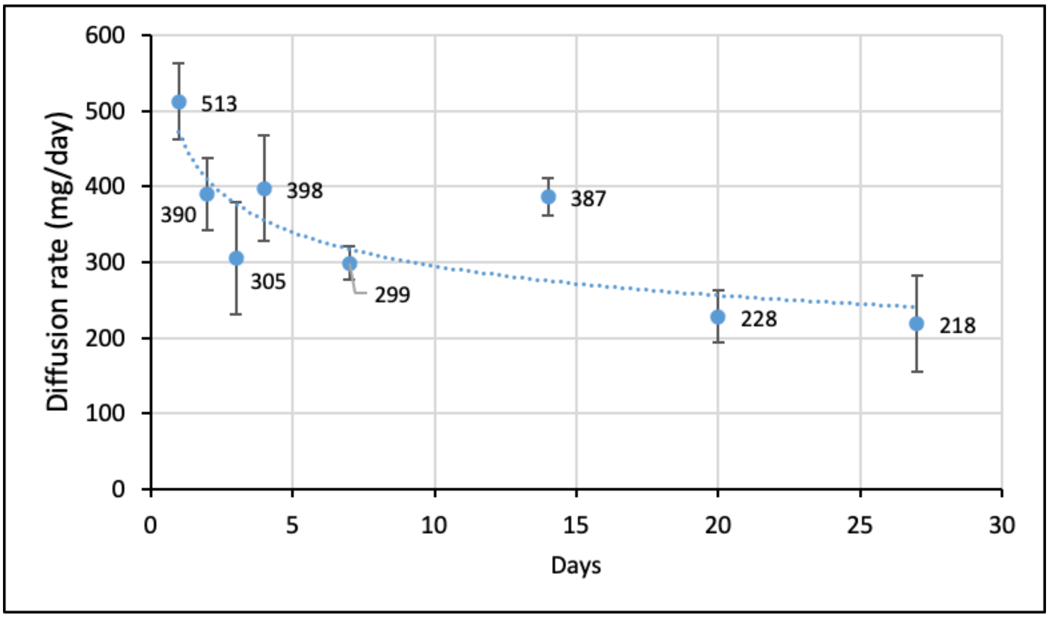

Figure 1. Diffusion rate of the denatured ethanol lures.

Mean daily diffusion rate (N=5) of ethanol from polythene Ziplock bags (12 x 8 cm x 50µ) containing 50 ml of denatured ethanol (ethanol 95% + 2-butanone <1% + propane-2-ol <1%) impregnating four 10 x 7 cm pieces of viscose tissue, and placed in a wind tunnel with a 0.05 m/sec airflow at 20°C. The mean diffusion rate during the first two weeks varied between 500 and 300 mg/d.

Table 1. Catches of *Ips typographus*, *Pityogenes chalcographus*, *Trypodendron lineatum*, *T. domesticum* and *T. signatum* in each type of trap at each date of servicing.

Line	Type of trap	Attractant	Date of servicing	*I. typographus*	*P. chalcographus*	*T. lineatum*	*T. domesticum*	*T. signatum*
1	fan-trap A	Lineatin + EtOH	3/30/2023	0	0	0	0	0
1	fan-trap B	Lineatin + EtOH	3/30/2023	0	0	0	0	1
1	fan-trap C	Lineatin + EtOH	3/30/2023	0	0	0	0	0
1	fan-trap D	Lineatin + EtOH	3/30/2023	0	0	0	0	0
1	cross-trap	Lineatin + EtOH	3/30/2023	0	0	0	0	0
2	fan-trap A	Lineatin + EtOH	3/30/2023	0	0	0	0	0
2	fan-trap B	Lineatin + EtOH	3/30/2023	0	0	0	0	0
2	fan-trap C	Lineatin + EtOH	3/30/2023	0	0	0	1	0
2	fan-trap D	Lineatin + EtOH	3/30/2023	0	0	0	0	0
2	cross-trap	Lineatin + EtOH	3/30/2023	0	0	0	0	0
3	fan-trap A	Lineatin + EtOH	3/30/2023	0	0	0	0	0
3	fan-trap B	Lineatin + EtOH	3/30/2023	0	0	0	0	0
3	fan-trap C	Lineatin + EtOH	3/30/2023	0	0	0	0	0
3	fan-trap D	Lineatin + EtOH	3/30/2023	0	0	0	0	0
3	cross-trap	Lineatin + EtOH	3/30/2023	0	0	0	0	0
4	fan-trap A	Lineatin + EtOH	3/30/2023	0	0	0	1	0
4	fan-trap B	Lineatin + EtOH	3/30/2023	0	0	0	0	0
4	fan-trap C	Lineatin + EtOH	3/30/2023	0	0	0	0	0
4	fan-trap D	Lineatin + EtOH	3/30/2023	0	0	0	0	0
4	cross-trap	Lineatin + EtOH	3/30/2023	0	0	0	0	0
5	fan-trap A	Lineatin + EtOH	3/30/2023	0	0	0	0	0
5	fan-trap B	Lineatin + EtOH	3/30/2023	0	0	0	0	0
5	fan-trap C	Lineatin + EtOH	3/30/2023	0	0	0	0	0
5	fan-trap D	Lineatin + EtOH	3/30/2023	0	0	0	0	0
5	cross-trap	Lineatin + EtOH	3/30/2023	0	0	0	0	0
1	fan-trap A	Lineatin + EtOH	4/13/2023	0	0	8	9	23
1	fan-trap B	Lineatin + EtOH	4/13/2023	0	0	71	7	19
1	fan-trap C	Lineatin + EtOH	4/13/2023	0	0	45	10	15
1	fan-trap D	Lineatin + EtOH	4/13/2023	0	0	33	9	15
1	cross-trap	Lineatin + EtOH	4/13/2023	0	0	119	36	30
2	fan-trap A	Lineatin + EtOH	4/13/2023	0	0	4	6	8
2	fan-trap B	Lineatin + EtOH	4/13/2023	0	0	8	4	8
2	fan-trap C	Lineatin + EtOH	4/13/2023	0	0	10	0	7
2	fan-trap D	Lineatin + EtOH	4/13/2023	0	0	23	9	12
2	cross-trap	Lineatin + EtOH	4/13/2023	NA	NA	NA	NA	NA
3	fan-trap A	Lineatin + EtOH	4/13/2023	0	0	4	1	4
3	fan-trap B	Lineatin + EtOH	4/13/2023	0	0	7	5	12
3	fan-trap C	Lineatin + EtOH	4/13/2023	0	0	15	2	20
3	fan-trap D	Lineatin + EtOH	4/13/2023	0	0	26	4	8
3	cross-trap	Lineatin + EtOH	4/13/2023	0	0	61	26	64
4	fan-trap A	Lineatin + EtOH	4/13/2023	0	0	4	22	12
4	fan-trap B	Lineatin + EtOH	4/13/2023	0	0	14	5	24
4	fan-trap C	Lineatin + EtOH	4/13/2023	0	0	0	0	0
4	fan-trap D	Lineatin + EtOH	4/13/2023	0	0	43	12	50
4	cross-trap	Lineatin + EtOH	4/13/2023	0	0	48	16	74
5	fan-trap A	Lineatin + EtOH	4/13/2023	0	0	5	2	1
5	fan-trap B	Lineatin + EtOH	4/13/2023	0	0	48	11	46
5	fan-trap C	Lineatin + EtOH	4/13/2023	0	0	30	3	36
5	fan-trap D	Lineatin + EtOH	4/13/2023	0	0	36	9	49
5	cross-trap	Lineatin + EtOH	4/13/2023	0	0	136	20	166

Caiti et al. - Supplementary material

Line	Type of trap	Attractant	Date of servicing	*I. typographus*	*P. chalcographus*	*T. lineatum*	*T. domesticum*	*T. signatum*
1	fan-trap A	Lineatin + EtOH	4/27/2023	0	0	2	1	3
1	fan-trap B	Lineatin + EtOH	4/27/2023	0	0	1	1	1
1	fan-trap C	Lineatin + EtOH	4/27/2023	0	0	7	0	9
1	fan-trap D	Lineatin + EtOH	4/27/2023	0	0	7	1	12
1	cross-trap	Lineatin + EtOH	4/27/2023	0	0	28	4	27
2	fan-trap A	Lineatin + EtOH	4/27/2023	0	0	3		4
2	fan-trap B	Lineatin + EtOH	4/27/2023	0	0	4	1	4
2	fan-trap C	Lineatin + EtOH	4/27/2023	0	0	3	1	3
2	fan-trap D	Lineatin + EtOH	4/27/2023	0	0	10	2	4
2	cross-trap	Lineatin + EtOH	4/27/2023	0	0	23	2	11
3	fan-trap A	Lineatin + EtOH	4/27/2023	0	0	3	0	2
3	fan-trap B	Lineatin + EtOH	4/27/2023	0	0	4	3	2
3	fan-trap C	Lineatin + EtOH	4/27/2023	0	0	5	2	2
3	fan-trap D	Lineatin + EtOH	4/27/2023	NA	NA	NA	NA	NA
3	cross-trap	Lineatin + EtOH	4/27/2023	0	0	39	5	21
4	fan-trap A	Lineatin + EtOH	4/27/2023	0	0	5	2	0
4	fan-trap B	Lineatin + EtOH	4/27/2023	0	0	11	1	1
4	fan-trap C	Lineatin + EtOH	4/27/2023	0	0	2	0	0
4	fan-trap D	Lineatin + EtOH	4/27/2023	0	0	33	5	14
4	cross-trap	Lineatin + EtOH	4/27/2023	0	0	24	3	15
5	fan-trap A	Lineatin + EtOH	4/27/2023	0	0	1	1	2
5	fan-trap B	Lineatin + EtOH	4/27/2023	0	0	21	4	8
5	fan-trap C	Lineatin + EtOH	4/27/2023	NA	NA	NA	NA	NA
5	fan-trap D	Lineatin + EtOH	4/27/2023	0	0	17	3	15
5	cross-trap	Lineatin + EtOH	4/27/2023	0	0	46	3	31
1	fan-trap A	Lineatin + EtOH	5/16/2023	0	12	47	1	29
1	fan-trap B	Lineatin + EtOH	5/16/2023	0	10	90	2	46
1	fan-trap C	Lineatin + EtOH	5/16/2023	0	0	44	0	36
1	fan-trap D	Lineatin + EtOH	5/16/2023	0	3	90	1	54
1	cross-trap	Lineatin + EtOH	5/16/2023	2	51	249	3	160
2	fan-trap A	Lineatin + EtOH	5/16/2023	0	4	42	1	45
2	fan-trap B	Lineatin + EtOH	5/16/2023	0	8	40	1	56
2	fan-trap C	Lineatin + EtOH	5/16/2023	0	7	61	2	48
2	fan-trap D	Lineatin + EtOH	5/16/2023	0	3	64	0	58
2	cross-trap	Lineatin + EtOH	5/16/2023	0	25	93	2	131
3	fan-trap A	Lineatin + EtOH	5/16/2023	0	2	39	1	47
3	fan-trap B	Lineatin + EtOH	5/16/2023	0	2	56	2	82
3	fan-trap C	Lineatin + EtOH	5/16/2023	0	5	71	0	77
3	fan-trap D	Lineatin + EtOH	5/16/2023	0	6	73	1	97
3	cross-trap	Lineatin + EtOH	5/16/2023	0	11	151	2	149
4	fan-trap A	Lineatin + EtOH	5/16/2023	NA	NA	NA	NA	NA
4	fan-trap B	Lineatin + EtOH	5/16/2023	0	0	58	2	42
4	fan-trap C	Lineatin + EtOH	5/16/2023	1	11	6	0	5
4	fan-trap D	Lineatin + EtOH	5/16/2023	0	9	63	2	63
4	cross-trap	Lineatin + EtOH	5/16/2023	0	15	83	0	104
5	fan-trap A	Lineatin + EtOH	5/16/2023	0	4	49	1	60
5	fan-trap B	Lineatin + EtOH	5/16/2023	0	5	55	0	65
5	fan-trap C	Lineatin + EtOH	5/16/2023	1	25	113	1	110
5	fan-trap D	Lineatin + EtOH	5/16/2023	0	5	58	0	91
5	cross-trap	Lineatin + EtOH	5/16/2023	0	7	109	1	137
1	fan-trap A	*Cis*-verbenol + methyl-butenol	5/25/2023	114	7	0	0	1
1	fan-trap B	*Cis*-verbenol + methyl-butenol	5/25/2023	203	4	0	0	0
1	fan-trap C	*Cis*-verbenol + methyl-butenol	5/25/2023	471	7	0	0	0

Caiti et al. - Supplementary material

Line	Type of trap	Attractant	Date of servicing	*I. typographus*	*P. chalcographus*	*T. lineatum*	*T. domesticum*	*T. signatum*
1	fan-trap D	*Cis*-verbenol + methyl-butenol	5/25/2023	492	16	0	0	0
1	cross-trap	*Cis*-verbenol + methyl-butenol	5/25/2023	2695	63	2	0	3
2	fan-trap A	*Cis*-verbenol + methyl-butenol	5/25/2023	114	10	0	0	0
2	fan-trap B	*Cis*-verbenol + methyl-butenol	5/25/2023	146	4	1	0	0
2	fan-trap C	*Cis*-verbenol + methyl-butenol	5/25/2023	115	5	0	0	1
2	fan-trap D	*Cis*-verbenol + methyl-butenol	5/25/2023	261	16	0	0	1
2	cross-trap	*Cis*-verbenol + methyl-butenol	5/25/2023	716	31	5	1	6
3	fan-trap A	*Cis*-verbenol + methyl-butenol	5/25/2023	166	1	0	0	0
3	fan-trap B	*Cis*-verbenol + methyl-butenol	5/25/2023	158	7	0	0	0
3	fan-trap C	*Cis*-verbenol + methyl-butenol	5/25/2023	257	17	1	0	1
3	fan-trap D	*Cis*-verbenol + methyl-butenol	5/25/2023	198	6	0	0	0
3	cross-trap	*Cis*-verbenol + methyl-butenol	5/25/2023	372	16	0	0	2
4	fan-trap A	*Cis*-verbenol + methyl-butenol	5/25/2023	83	5	0	0	0
4	fan-trap B	*Cis*-verbenol + methyl-butenol	5/25/2023	289	2	0	0	0
4	fan-trap C	*Cis*-verbenol + methyl-butenol	5/25/2023	269	8	0	0	0
4	fan-trap D	*Cis*-verbenol + methyl-butenol	5/25/2023	601	11	0	0	0
4	cross-trap	*Cis*-verbenol + methyl-butenol	5/25/2023	1108	23	0	0	1
5	fan-trap A	*Cis*-verbenol + methyl-butenol	5/25/2023	806	11	0	0	0
5	fan-trap B	*Cis*-verbenol + methyl-butenol	5/25/2023	223	5	0	0	0
5	fan-trap C	*Cis*-verbenol + methyl-butenol	5/25/2023	174	7	0	0	0
5	fan-trap D	*Cis*-verbenol + methyl-butenol	5/25/2023	635	18	0	0	0
5	cross-trap	*Cis*-verbenol + methyl-butenol	5/25/2023	1141	49	0	0	2
1	fan-trap A	*Cis*-verbenol + methyl-butenol	7/06/2023	89	5	0	0	0
1	fan-trap B	*Cis*-verbenol + methyl-butenol	7/06/2023	211	22	0	0	0
1	fan-trap C	*Cis*-verbenol + methyl-butenol	7/06/2023	298	9	1	0	1
1	fan-trap D	*Cis*-verbenol + methyl-butenol	7/06/2023	355	35	0	0	0
1	cross-trap	*Cis*-verbenol + methyl-butenol	7/06/2023	977	43	1	0	0
2	fan-trap A	*Cis*-verbenol + methyl-butenol	7/06/2023	83	8	0	0	0
2	fan-trap B	*Cis*-verbenol + methyl-butenol	7/06/2023	157	12	2	0	0
2	fan-trap C	*Cis*-verbenol + methyl-butenol	7/06/2023	222	13	0	0	0
2	fan-trap D	*Cis*-verbenol + methyl-butenol	7/06/2023	234	19	0	0	0
2	cross-trap	*Cis*-verbenol + methyl-butenol	7/06/2023	796	55	1	0	1
3	fan-trap A	*Cis*-verbenol + methyl-butenol	7/06/2023	106	9	0	0	0
3	fan-trap B	*Cis*-verbenol + methyl-butenol	7/06/2023	133	22	0	0	0
3	fan-trap C	*Cis*-verbenol + methyl-butenol	7/06/2023	364	49	0	0	0
3	fan-trap D	*Cis*-verbenol + methyl-butenol	7/06/2023	369	30	0	0	0
3	cross-trap	*Cis*-verbenol + methyl-butenol	7/06/2023	853	43	0	0	0
4	fan-trap A	*Cis*-verbenol + methyl-butenol	7/06/2023	256	37	0	0	0
4	fan-trap B	*Cis*-verbenol + methyl-butenol	7/06/2023	200	5	0	0	0
4	fan-trap C	*Cis*-verbenol + methyl-butenol	7/06/2023	329	0	0	0	0
4	fan-trap D	*Cis*-verbenol + methyl-butenol	7/06/2023	477	7	0	0	0
4	cross-trap	*Cis*-verbenol + methyl-butenol	7/06/2023	918	143	0	0	0
5	fan-trap A	*Cis*-verbenol + methyl-butenol	7/06/2023	149	20	0	0	0
5	fan-trap B	*Cis*-verbenol + methyl-butenol	7/06/2023	437	30	0	0	0
5	fan-trap C	*Cis*-verbenol + methyl-butenol	7/06/2023	243	25	0	0	0
5	fan-trap D	*Cis*-verbenol + methyl-butenol	7/06/2023	2192	78	0	0	1
5	cross-trap	*Cis*-verbenol + methyl-butenol	7/06/2023	975	82	0	0	0

Table 2. Catches and density (individuals/cm^2) of *Anisandrus dispar*, *Trypodendron lineatum* + *T. signatum* and *T. domesticum* at increasing distances from the attractants in each half-quadrant of the glue trap during the period 15/05/2002 to 23/05/2002.

Half-quadrant	Distance from the center (cm)	Catches of *A. dispar*	Density of *A. dispar*	Catches of *T. domesticum*	Density of *T. domesticum*	Catches of *T. signatum* + *T. lineatum*	Density of *T. signatum* + *T. lineatum*
1	10	0	0.000	0	0.000	0	0.000
1	15	0	0.000	2	0.041	1	0.020
1	20	1	0.015	0	0.000	1	0.015
1	25	3	0.034	0	0.000	0	0.000
1	30	7	0.065	3	0.028	0	0.000
1	35	9	0.071	0	0.000	0	0.000
1	40	13	0.088	1	0.007	1	0.007
1	45	21	0.126	2	0.012	2	0.012
2	50	24	0.129	11	0.059	4	0.021
2	10	1	0.034	0	0.000	0	0.000
2	15	0	0.000	1	0.020	0	0.000
2	20	2	0.029	0	0.000	0	0.000
2	25	2	0.023	0	0.000	0	0.000
2	30	9	0.083	4	0.037	0	0.000
2	35	4	0.031	3	0.024	0	0.000
2	40	6	0.041	4	0.027	1	0.007
2	45	12	0.072	2	0.012	0	0.000
2	50	20	0.107	2	0.011	2	0.011
3	10	0	0.000	0	0.000	1	0.034
3	15	0	0.000	0	0.000	0	0.000
3	20	0	0.000	0	0.000	0	0.000
3	25	0	0.000	1	0.011	0	0.000
3	30	4	0.037	0	0.000	1	0.009
3	35	4	0.031	0	0.000	1	0.008
3	40	1	0.007	2	0.014	0	0.000
3	45	3	0.018	1	0.006	2	0.012
3	50	5	0.027	1	0.005	1	0.005
4	10	0	0.000	0	0.000	0	0.000
4	15	0	0.000	3	0.061	0	0.000
4	20	0	0.000	0	0.000	0	0.000
4	25	1	0.011	0	0.000	0	0.000
4	30	3	0.028	0	0.000	2	0.019
4	35	0	0.000	0	0.000	1	0.008
4	40	1	0.007	0	0.000	0	0.000
4	45	4	0.024	1	0.006	1	0.006
4	50	4	0.021	0	0.000	1	0.005
5	10	0	0.000	0	0.000	0	0.000
5	15	1	0.020	0	0.000	0	0.000
5	20	0	0.000	0	0.000	0	0.000
5	25	3	0.034	0	0.000	0	0.000
5	30	0	0.000	0	0.000	0	0.000
5	35	1	0.008	0	0.000	0	0.000
5	40	2	0.014	0	0.000	2	0.014
5	45	0	0.000	0	0.000	0	0.000
5	50	4	0.021	3	0.016	1	0.005
6	10	0	0.000	0	0.000	0	0.000
6	15	0	0.000	0	0.000	0	0.000

Half-quadrant	Distance from the center (cm)	Catches of *A. dispar*	Density of *A. dispar*	Catches of *T. domesticum*	Density of *T. domesticum*	Catches of *T. signatum + T. lineatum*	Density of *T. signatum + T. lineatum*
6	20	2	0.029	1	0.015	0	0.000
6	25	3	0.034	2	0.023	0	0.000
6	30	1	0.009	1	0.009	0	0.000
6	35	5	0.039	1	0.008	0	0.000
6	40	5	0.034	0	0.000	2	0.014
6	45	6	0.036	1	0.006	1	0.006
6	50	10	0.054	4	0.021	1	0.005
7	10	0	0.000	0	0.000	0	0.000
7	15	0	0.000	0	0.000	1	0.020
7	20	4	0.058	0	0.000	1	0.015
7	25	2	0.023	0	0.000	1	0.011
7	30	5	0.046	2	0.019	1	0.009
7	35	10	0.078	2	0.016	1	0.008
7	40	9	0.061	2	0.014	2	0.014
7	45	7	0.042	2	0.012	0	0.000
7	50	19	0.102	3	0.016	3	0.016
8	10	0	0.000	0	0.000	0	0.000
8	15	0	0.000	0	0.000	0	0.000
8	20	2	0.029	1	0.015	1	0.015
8	25	0	0.000	0	0.000	0	0.000
8	30	4	0.037	1	0.009	3	0.028
8	35	6	0.047	1	0.008	0	0.000
8	40	11	0.075	1	0.007	2	0.014
8	45	8	0.048	3	0.018	2	0.012
8	50	27	0.145	3	0.016	3	0.016

Table 3. Details of the multiple comparisons (p-value)

Ips typographus				
Interception Area (cm^2)	310	536	832.5	1200
536	0.310	-	-	-
832.5	0.278	0.310	-	-
1200	0.111	0.079	0.278	-
4500	0.026	0.026	0.026	0.159
Pairwise comparisons using Wilcoxon rank sum exact test with the Benjamini-Hochberg adjustment				

Pityogenes chlacographus				
Interception Area (cm^2)	310	536	832.5	1200
536	0.999	-	-	-
832.5	0.969	0.975	-	-
1200	0.575	0.596	0.903	-
4500	0.00005	0.00005	0.0002	0.001
Tukey multiple comparisons of means 95% family-wise confidence level				

Trypodendron signatum				
Interception Area (cm^2)	310	536	832.5	1200
536	0.713	-	-	-
832.5	0.879	0.997	-	-
1200	0.227	0.888	0.726	-
4500	0.00003	0.0004	0.0002	0.003
Tukey multiple comparisons of means 95% family-wise confidence level				

Trypodendron lineatum				
Interception Area (cm^2)	310	536	832.5	1200
536	0.063	-	-	-
832.5	0.215	1.00	-	-
1200	0.040	0.467	0.217	-
4500	0.040	0.093	0.053	0.063
Pairwise comparisons using Wilcoxon rank sum exact test with the Benjamini-Hochberg adjustment				

Trypodendron domesticum				
Interception Area (cm^2)	310	536	832.5	1200
536	0.75	-	-	-
832.5	0.45	0.18	-	-
1200	0.45	0.45	0.10	-
4500	0.25	0.25	0.10	0.25
Pairwise comparisons using Wilcoxon rank sum exact test with the Benjamini-Hochberg adjustment				

Table 4. Comparisons with the literature – summary data

**Our data** (Gedinne 2023) *Ips typographus*			
**Trap size**	**Total catches** (N= 5)	**Ratio catches**	**Ratio sizes**
310	1,966	0.08	1.0
536	2,157	0.09	1.7
832,5	2,744	0.12	2.7
1200	5,814	0.25	3.9
4500	10,553	0.45	14.5
**Total**	**23,234**		

**Our data** (Gedinne 2023) *Pityogenes chalcographus*			
**Trap size**	**Total catches** (N= 5)	**Ratio catches**	**Ratio sizes**
310	135	0.10	1.0
536	138	0.10	1.7
832,5	188	0.14	2.7
1200	262	0.19	3.9
4500	657	0.48	14.5
**Total**	**1,380**		

**Our data** (Gedinne 2023) *Trypodendron lineatum*			
**Trap size**	**Total catches** (N= 5)	**Ratio catches**	**Ratio sizes**
310	216	0.04	1.0
536	491	0.09	1.7
832.5	414	0.07	2.7
1,200	576	0.10	3.9
4,500	1,218	0.22	14.5
**Total**	**2,915**		

**Our data** (Gedinne 2023) *Trypodendron signatum*			
**Trap size**	**Total catches** (N= 5)	**Ratio catches**	**Ratio sizes**
310	241	0.04	1.0
536	417	0.07	1.7
832.5	371	0.07	2.7
1,200	544	0.10	3.9
4,500	1,135	0.20	14.5
**Total**	**2,708**		

**Brar et al 2012** *Xyleborus glabratus*			
**Funnels**	**Mean catches** (N= 5)	**Ratio catches**	**Ratio sizes**
4	4.1	0.18	1.0
8	7	0.30	2.0
12	6	0.26	3.0
16	6.3	0.27	4.0
**Total**	**23.4**		

**Francese et al 2013** *Agrilus planipennis*			
**Funnels**	**Mean catches** (N= 15)	**Ratio catches**	**Ratio sizes**
4	12.2	0.05	1.0
8	48.2	0.19	2.0
12	98.2	0.38	3.0
16	98.9	0.38	4.0
**Total**	**257.5**		

Hoover et al 2000<br>*Trypodendron lineatum* males<br>(four trap lengths, each with equal release rate)			
Funnels	Mean catches (N= 10)	Ratio catches	Ratio sizes
4	341.1	0.08	1.0
8	733.4	0.17	2.0
12	1,345.5	0.32	3.0
16	1,782.2	0.42	4.0
Total	**4,202.2**		

Hoover et al .2000<br>*Trypodendron lineatum* females<br>(four trap lengths, each with equal release rate)			
Funnels	Mean catches (N= 10)	Ratio catches	Ratio sizes
4	210.3	0.08	1.0
8	457.1	0.16	2.0
12	876.1	0.32	3.0
16	1,233.5	0.44	4.0
Total	**2,777**		

Lindgren et al. 2000<br>*Trypodendron rufitarsus* males<br>(four trap lengths, each with equal release rate)			
Funnels	Mean catches (N= 10)	Ratio catches	Ratio sizes
4	17.4	0.07	1.0
8	40.5	0.17	2.0
12	85.7	0.36	3.0
16	93.5	0.39	4.0
Total	**237.1**		

Lindgren et al. 2000<br>*Trypodendron rufitarsus* females<br>(four trap lengths, each with equal release rate)			
Funnels	Mean catches (N= 10)	Ratio catches	Ratio sizes
4	29.2	0.08	1.0
8	69.8	0.19	2.0
12	103.7	0.28	3.0
16	163.9	0.45	4.0
Total	**366.6**		

References for Table 4

Brar, G. S., Capinera, J. L., McLean, S., Kendra, P. E., Ploetz, R. C., & Peña, J. E. (2012). Effect of trap size, trap height and age of lure on sampling *Xyleborus glabratus* (Coleoptera: Curculionidae: Scolytinae), and its flight periodicity and seasonality. *Flo. Entom.*, ***95***(4), 1003-1011. https://doi.org/10.1653/024.095.0428

Francese, J. A., Rietz, M. L., & Mastro, V. C. (2013). Optimization of multifunnel traps for emerald ash borer (Coleoptera: Buprestidae): influence of size, trap coating, and color. *J. Econ. Entomol.*, ***106***(6), 2415-2423. https://doi.org/10.1603/EC13014

Hoover, S. E. R., Lindgren, B. S., Keeling, C. I., & Slessor, K. N. (2000). Enantiomer preference of *Trypodendron lineatum* and effect of pheromone dose and trap length on response to lineatin-baited traps in interior British Columbia. *J. Chem. Ecol.*, *26*, 667-677. https://doi.org/10.1023/A:1005476105114

Lindgren, B. S., Hoover, S. E. R., MacIsaac, A. M., Keeling, C. I., & Slessor, K. N. (2000). Lineatin enantiomer preference, flight periods, and effect of pheromone concentration and trap length on three sympatric species of *Trypodendron* (Coleoptera: Scolytidae). *Can. Entomol.*, *132*(6), 877-887. https://doi.org/10.4039/Ent132877-6

Trends in the responses to increasing trap sizes (range: 310 – 832.5 cm^2; 310 – 1,200 cm^2)

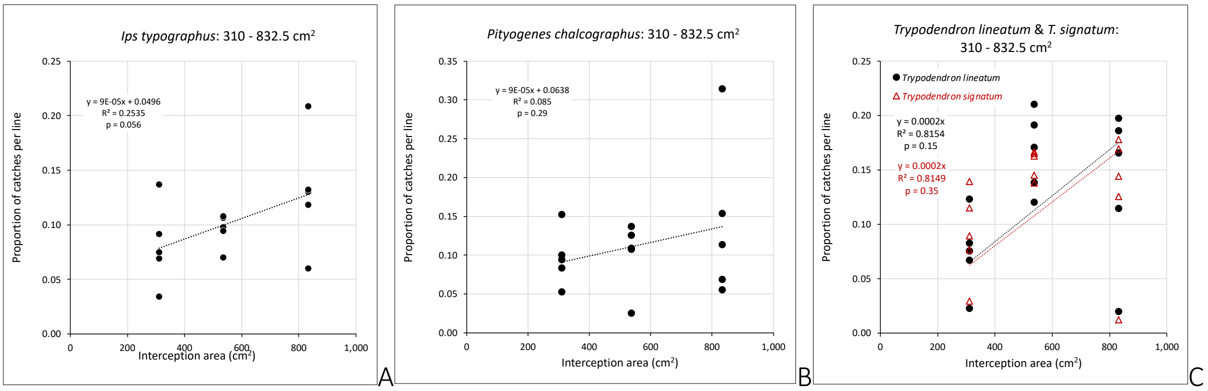

Figure 2. Trends in the responses to increasing trap sizes (range: 310 – 832.5 cm^2).
A: *Ips typographus* (see Fig. 6A box 1 in main text); **B**: *Pityogenes chalcographus* (see Fig. 6E box 1);
C: *Trypodendron lineatum* & *T. signatum* (see Fig. 6D box 1)

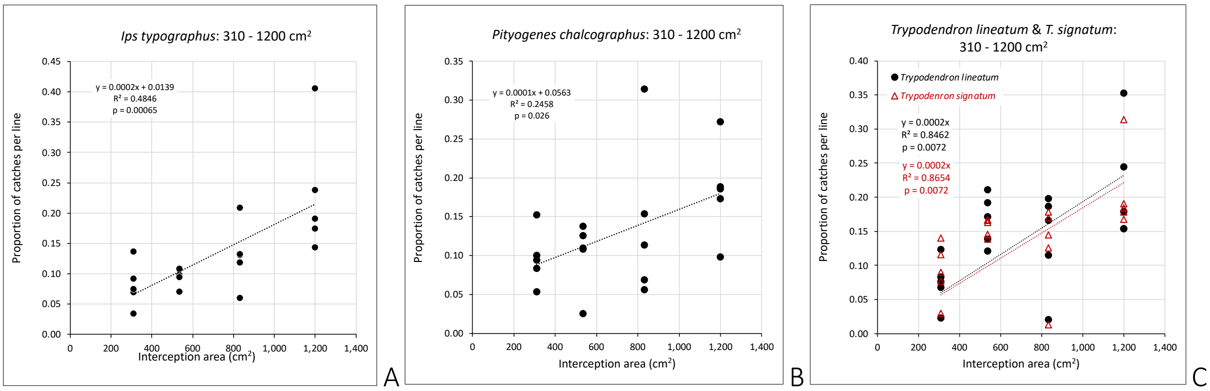

Figure 3. Trends in the responses to increasing trap sizes (range: 310 – 1,200 cm^2).
A: *Ips typographus* (see Fig. 6A box 2 in main text); **B**: *Pityogenes chalcographus* (see Fig. 6E box 2);
C: *Trypodendron lineatum* & *T. signatum* (see Fig. 6D box 2)
